# Emerging challenges in understanding trypanosome antigenic variation

**DOI:** 10.1042/ETLS20170104

**Published:** 2017-12-22

**Authors:** Richard McCulloch, Christina A. Cobbold, Luisa Figueiredo, Andrew Jackson, Liam J. Morrison, Monica R. Mugnier, Nina Papavasiliou, Achim Schnaufer, Keith Matthews

**Affiliations:** 1 The Wellcome Centre for Molecular Parasitology, University of Glasgow, 120 University Place, Glasgow G12 8TA, U.K.; 2 School of Mathematics and Statistics, University of Glasgow, University Place, Glasgow G12 8QS, U.K.; 3 Instituto de Medicina Molecular, Avenida Professor Egas Moniz, Lisboa 1649-028, Portugal; 4 Institute of Infection and Global Health, Department of Infection Biology, University of Liverpool, 146 Brownlow Hill, Liverpool L3 5RF, U.K.; 5 The Roslin Institute, Royal (Dick) School of Veterinary Studies, Division of Infection and Immunity, University of Edinburgh, Easter Bush, Midlothian EH25 9RG, U.K.; 6 Department of Molecular Microbiology and Immunology, Johns Hopkins Bloomberg School of Public Health, Baltimore, MD, U.S.A.; 7 Immune Diversity (D150), Division Immunology, German Cancer Research Center, Im Neuenheimer Feld 280, Heidelberg 69120, Germany; 8 Institute for Immunology and Infection Research, School of Biological Sciences, University of Edinburgh, Ashworth Laboratories, Charlotte Auerbach Road, Edinburgh EH9 3FL, U.K.

**Keywords:** antigenic variaton, host–pathogen interactions, trypanosomes

## Abstract

Many pathogens evade host immunity by periodically changing the proteins they express on their surface — a phenomenon termed antigenic variation. An extreme form of antigenic variation, based around switching the composition of a variant surface glycoprotein (VSG) coat, is exhibited by the African trypanosome *Trypanosoma brucei*, which causes human disease. The molecular details of VSG switching in *T. brucei* have been extensively studied over the last three decades, revealing in increasing detail the machinery and mechanisms by which VSG expression is controlled and altered. However, several key components of the models of *T. brucei* antigenic variation that have emerged have been challenged through recent discoveries. These discoveries include new appreciation of the importance of gene mosaics in generating huge levels of new VSG variants, the contributions of parasite development and body compartmentation in the host to the infection dynamics and, finally, potential differences in the strategies of antigenic variation and host infection used by the crucial livestock trypanosomes *T. congolense* and *T. vivax*. This review will discuss all these observations, which raise questions regarding how secure the existing models of trypanosome antigenic variation are. In addition, we will discuss the importance of continued mathematical modelling to understand the purpose of this widespread immune survival process.

## Introduction

**Figure 1.**
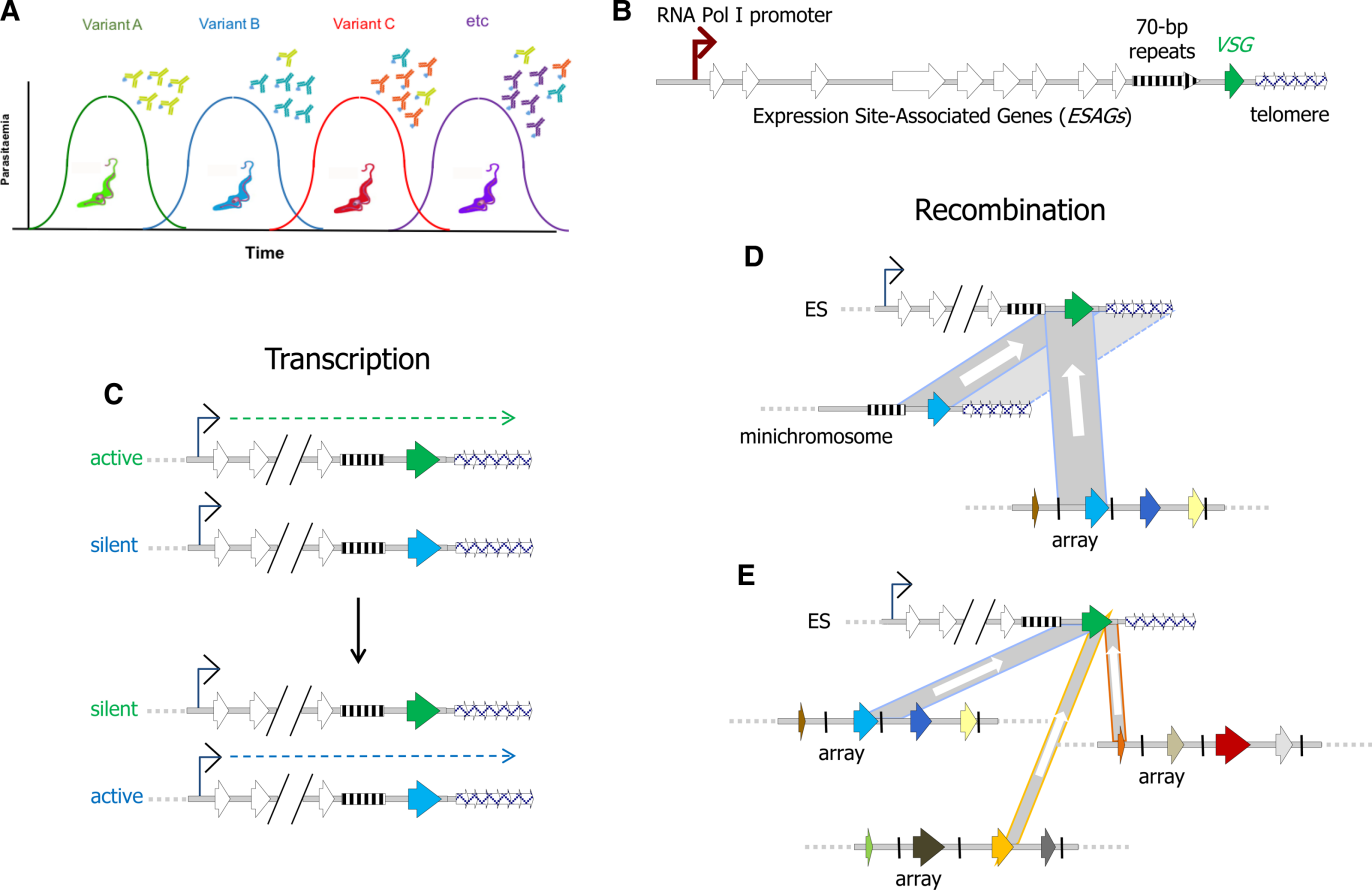
Antigenic variation in *Trypanosoma brucei*. (**A**) A view of a trypanosome infection profile, where progressive waves of parasitaemia are composed of trypanosome populations with antigenically distinct VSG coats. For simplicity, each wave is shown to contain a population expressing a single VSG coat (different coloured cells; variants A, B, C, etc.), which results in antibodies against that variant; however, normally many parasites with different VSGs are found per wave. (**B**) A depiction of a VSG ES that is used when *T. brucei* is found in the mammal. Multiple expression site-associated genes (ESAGs; white arrows) are co-expressed with the VSG (green arrow), which is adjacent to the telomere and downstream from 70 bp repeats. Multigenic transcription across the ES is derived from an RNA Pol I promoter. (**C**) Transcriptional VSG coat switching, where transcription (green arrow) from the single active VSG ES is silenced, and transcription is up-regulated across a previously silent VSG ES (blue arrow and VSG). (**D** and **E**) VSG coat switching by recombination, of which two forms of gene conversion are shown. In one reaction (**D**), an intact, silent VSG gene copy (blue arrow) in a minichromosome, sub-telomeric VSG array or silent ES (not shown) is recombined into the active ES based on upstream and downstream sequence homology. In the second reaction (**E**), segmental gene conversion occurs between multiple silent VSGs and VSG pseudogenes (yellow and orange arrows) in the VSG arrays to form a novel, patchwork VSG mosaic; for simplicity, this event is shown to occur in the VSG ES, but the location of the mosaic assembly is unknown.

Pathogens face a key challenge for their long-term survival, ensuring transmission between hosts. An approach adopted frequently by pathogens to enhance transmission is to persist as chronic infections, increasing the likelihood of transfer to a new host. To maintain a chronic infection in mammals, the substantial obstacle of prolonged exposure to the acquired and innate immune systems must be overcome. One widespread strategy to avoid immune elimination, found in viruses, bacteria, fungi and protozoans, is antigenic variation, where exposed pathogen antigens are periodically replaced. In all cases, the generation of host immune effectors that recognise an exposed antigen will eliminate most pathogens, but by switching the expressed surface antigen, a subpopulation of the infecting pathogen avoids host recognition and killing. Repeated rounds of immune recognition, killing and outgrowth of switched subpopulations can lead to waves of increasing and decreasing pathogen loads (Figure 1A). Antigenic variation has shared features in pathogens including *Neisseria*, *Borrelia*, *Anaplasma*, *Giardia*, *Plasmodium*, *Babesia* and African trypanosomes [1–5], such as gene expression control to ensure a single antigen is expressed in a single cell at one time, a repertoire of antigen genes and a means to elicit a switch in the singularly expressed antigen gene among the repertoire. However, because antigenic variation evolved separately in these organisms, the mechanisms dictating the shared features are highly diverse.

In African trypanosomes, antigenic variation involves switches in the expression of variant surface glycoprotein (VSG) [6,7], which is thought to form a dense protective coat across the whole parasite cell, shielding necessarily invariant antigens [8]. Loss of the VSG coat is lethal even in culture [9], underlining its importance to *Trypanosoma brucei* growth and survival in even the most forgiving environments. The genetic components of antigenic variation in *T. brucei* have been known for nearly 40 years [10]. Since then, sequencing the genome of *T. brucei* [11] allied to the development of a wide range of genetic tools (e.g. gene knockout, RNAi, overexpression and genome-wide screens) has provided effective strategies to perturb the system, which has revealed detailed information on the molecular basis of VSG expression switching. What has emerged is a system of remarkable mechanistic flexibility (Figure 1) and a potentially unparalleled capacity for new coat generation. Monoallelic expression, such that each *T. brucei* cell expresses a single VSG variant at one time, relies upon VSG genes only being expressed when present in specialised telomeric VSG expression sites (ESs). Unusually, the ESs are not transcribed by RNA Polymerase (Pol) II, but by RNA Pol I (Figure 1B). The *T. brucei* genome contains multiple ESs [12] and so one route to execute a VSG coat switch is to silence the actively transcribed ES and activate one previously silent ES (Figure 1C). A range of factors have been described that ensure singular ES expression [13–16], and a few have been implicated in the co-ordination of transcriptional switching [17–19].

Trypanosomes can also execute a VSG coat switch by genetic recombination (Figure 1D,E), and it is this strategy of gene rearrangement that maintains chronic infections. The *T. brucei* genome contains thousands of transcriptionally silent VSG genes [11,20,21], found as arrays in the sub-telomeres of the diploid megabase chromosome and at the telomeres of hundreds of minichromosomes [22]. Only a minority of the VSG archive is composed of functional VSGs [11,20,21], but these are preferentially activated early in infections [23] and extensive genetic analyses indicate that homologous recombination (HR), a general genome repair pathway, catalyses a VSG switch [24]. How this reaction is initiated is still being examined, but it seems likely the actively transcribed ES is preferentially targeted [25–30]. Later in infections, activation of VSG pseudogenes predominates, with VSG recombination operating by a potentially distinct route to that used for intact VSGs: highly flexible segmental gene conversion occurs (Figure 1E), which reassorts multiple VSGs using intra-open reading frame homology to yield novel patchwork ‘mosaic’ VSGs [21,31,32]. This reaction appears to be the key to chronic infections [33], but the nature of how it is executed is mysterious (see below).

**Figure 2.**
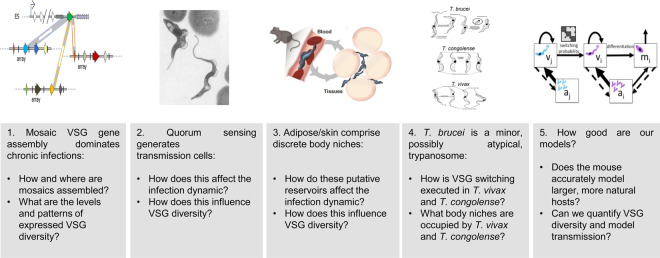
Emerging questions in trypanosome antigenic variation. Five areas of emerging trypanosome biology, and some questions that arise regarding antigenic variation are highlighted in the boxes. Each box is discussed in greater depth in the text of the article.

The burgeoning and ongoing dissection of antigenic variation has revealed a wealth of new biology, but several recent studies (Figure 2) suggest that our view of the nature and purpose of the process need to be reconsidered in the light of chronic infections, in terms of transmission, and with regard to how well the reactions described in *T. brucei* reflect what occurs in other trypanosome species. Below we summarise the emerging data and the questions raised regarding antigenic variation in trypanosomes.

## How dynamic is the expressed and genomic VSG repertoire, and how is VSG coat switching catalysed?

The textbook paradigm of each expressed VSG coat type being encoded by its own intact VSG gene, selected from an archive of such genes and activated one at a time by gene conversion (Figure 1), is now recognised as oversimplistic. VSG coat variants do not arise in a simple homogeneous procession. Instead, longitudinal sampling of the expressed VSG RNA repertoire during mice infections by targeted cloning [31] or RNAseq [32] confirmed predictions [23,34] that VSG variants arise semi-stochastically and revealed that each wave of parasitaemia comprises populations of trypanosomes expressing many distinct VSGs, with grossly differing abundance. Moreover, the flexibility of segmental VSG gene conversion predicts that the expressed repertoire hugely exceeds the genomic VSG archive [31,35,36]. The predominance of mosaic VSG formation later in infections raises a range of questions (Figure 2, Q.1). What is the true scale of expressed VSG diversity, and does antigen expression increase in complexity over time? Do lineages of related (but antigenically distinct) mosaic VSGs form during infections due to inherent differences in the activation rates of VSG (pseudo)genes, what proportions of such mosaics are antigenically distinct, and how does putative lineage diversity vary between infections?

Answering such questions will require further and bigger datasets spanning the great length of chronic infections, allied to robust mathematical modelling, not least to quantify expressed VSG diversity (see below). Beyond the impact of segmental VSG gene conversion on expressed diversity, we currently have no insights into the recombination pathway that directs the reaction. For instance, while it is clear that RAD51-catalysed recombination, guided by BRCA2 [37,38], acts to recombine intact VSGs, no study has evaluated the effect of loss of these factors on the extent and pattern of VSG diversity as infections progress, and so we cannot rule out that a non-HR reaction catalyses segmental gene conversion. In addition, whether novel mosaic VSG genes are assembled within the VSG ES, or whether the reaction occurs within the silent sub-telomeric VSG archive (with subsequent recombination into the VSG ES) is unknown. Changes in the VSG archive are known to cause variation in chromosome size between *T. brucei* isolates [39], but the nature of the underlying rearrangements and their link to VSG switching during chronic infections has not been evaluated. All these aspects of VSG switching and archive stability have implications for onward transmission.

## How does antigenic variation operate if most parasites are non-proliferative?

The infection dynamic of *T. brucei* does not simply derive from antigenic variation, but is critically shaped by the parasite's development in the bloodstream. Specifically, in each wave of parasitaemia, the parasites proliferate as ‘slender forms’ until a density threshold is reached, whereupon they differentiate to ‘stumpy forms’, adapted for transmission to the tsetse fly vector (Figure 2, Q.2). At least one pathway of this developmental transition represents a form of quorum-sensing generated by a parasite-derived signal, termed the ‘stumpy induction factor’ (SIF) [40,41]. Although SIF remains unidentified, its signal transduction pathway(s) has been dissected by genome-wide RNAi screening and targeted mutation analysis, identifying components, such as protein kinases and phosphatases, as well as gene regulators, whose loss renders the parasites unresponsive to SIF [42–47]. Cytological markers that distinguish slender and stumpy forms have allowed the quantification of each cell type throughout the course of infection [48]. In chronic mouse infections, this tool has been used to establish that irreversibly cell-cycle arrested stumpy forms predominate [49]. With only a subset of the parasite population (slender forms) proliferating, the frequency of productive antigenic variation, generated through DNA recombination, may be lower than expected and the outgrowth of new variants is inevitably restricted, raising questions about how differentiation and VSG switching interact to influence expressed VSG diversity [50]. Indeed, this may have wider implications for infection dynamics, since it has been shown that SIF can be exchanged between *T. congolense* and *T. brucei* [51], meaning co-infection dynamics may not be simple competitions between different infecting parasites.

## What is the contribution of extravascular tissues to antigenic variation?

Recent work has reshaped our view of *T. brucei* as being a parasite that lives and proliferates mainly in the bloodstream and lymphatic systems. While trypanosome movement between the blood and tissues has long been recognised, methods for the isolation and quantitation of parasites have revealed that a significant portion — perhaps a majority — of the population resides extravascularly in the adipose tissue [52] and skin [53] (Figure 2, Q.3). Both these sites may be immune-privileged [54], perhaps providing significant reservoirs for maintaining infections and facilitating transmission. While slender and stumpy forms of *T. brucei* can be found in the skin and adipose tissue, it has not yet been established if differentiation occurs in these tissues. Nonetheless, at least in the adipose tissue, the cells exhibit a distinct metabolism to circulating bloodstream form cells [52,55], indicating they represent a discrete, adapted population. Importantly, the rates and mechanisms of antigenic variation in these locations are unknown, as is the capacity for flux of variants between the blood and body niches, meaning existing strategies to evaluate expressed VSG diversity need to be revisited. Moreover, the ability of immune responses to select the enormous diversity of antigen types in circulation and in different body compartments, and thereby shape the infection dynamic, also requires mechanistic and population-scale analysis. Finally, the intravascular behaviour of at least one other *Trypanosoma* species, *T. congolense*, appears distinct from *T. brucei* [56,57], questioning whether intra-host infection dynamics are shared across African trypanosomes. Indeed, *T. equiperdum*, which is more closely related to *T. brucei* [58], may represent an extreme case of tissue tropism, where the bloodstream as a habitat has been substantially abandoned [59].

## How does antigenic variation operate in livestock trypanosome infections?

Broader questions relate to the widespread use of *T. brucei* in mice as the infection model. *T. brucei gambiense* and *T. brucei rhodesiense* are the causative agents of increasingly scarce human infections [60–63], which can be modelled in mice using *T. brucei brucei*, even though this subspecies is not human infective [64]. However, mice are an unnatural host, and the most significant impact associated with trypanosomiasis is that caused by livestock infections, which are predominantly caused by *T. congolense* and *T. vivax*, each being more prevalent and pathogenic in cattle than *T. brucei* [65]. Though both of these trypanosome species rely on VSG switching for immune evasion, unlike other bovine-infective *Trypanosoma* species [66], genome comparisons have revealed a much smaller proportion of VSG pseudogenes relative to *T. brucei*, differences in conserved coding and non-coding sequences that flank VSG loci relative to *T. brucei*, and distinct patterns of inferred recombination among VSGs relative to *T. brucei* [67] (Figure 2, Q.4). All these findings raise questions about the extent to which the mechanisms for VSG switching described in *T. brucei* apply to *T. vivax* and *T. congolense*. Indeed, a striking expansion in the number of functional BRC motifs in *T. brucei* BRCA2, which directs VSG recombination, is not found in the BRCA2 homologues of *T. congolense* and *T. vivax*, perhaps consistent with differing recombination routes for VSG switching or diversification [37,38]. Any underlying differences in VSG switch mechanism or usage will be most readily evaluated by comparing VSG expression diversity during chronic infections by the different trypanosomes, as well as by extrapolating targeted genetic mutants from *T. brucei* to *T. congolense* and *T. vivax*.

## Have we adequately modelled antigenic variation?

Experimental analysis of antigenic variation is critical, but frequently focuses on aspects of mechanism and biology that are amenable to genetic perturbation. Given the huge size of the *T. brucei* VSG archive, the realisation of high levels of expressed VSG diversity [largely due to segmental VSG (pseudo)gene conversion] and the emergence of differentiation and tissue compartmentalisation having an influence on the infection dynamics, it is clear that we must continue to evaluate our mechanistic understanding of antigenic variation with mathematical modelling (Figure 2, Q.5). Only modelling approaches have the capacity to integrate distinct empirical advances in such a way that we can evaluate the extent to which the biological processes discussed above interact to shape the overall infection dynamic, and to understand how this influences the expressed VSG diversity within and between species. Existing models that have used known infection parameters and VSG switch rates [34,68] may need to be re-examined in the light of the above emerging data. In addition, evaluation of the gene sequences associated with VSG gene conversion [69] needs to consider the potential for different homology requirements and recombination mechanisms during intact VSG gene conversion and segmental VSG (pseudo)gene conversion, as well the possibility that the reaction constraints and usage may differ between *Trypanosoma* species. Finally, though antigenic variation is a strategy to maintain chronic infections, it is very likely that it is also needed to allow pathogens to counter the emergence of herd immunity and thereby infect pre-infected hosts. Innovative modelling of VSG switching is needed to make testable predictions regarding how antigenic variation operates at the cellular, within-host and between-host scales [70], and ultimately how it contributes to pathogen transmission.

## Summary

- Understanding of the roles and mechanisms of mosaic VSG switching is incomplete.
- How infection dynamics and antigenic variation are shaped by density-dependent differentiation needs to be determined.
- The roles and impact of tissue compartmentalisation needs to be evaluated.
- Comparison must be made of antigenic variation in human and livestock trypanosomes.
- Continued modelling remains central to understanding antigenic variation.

## Abbreviations

ES: expression sites
HR: homologous recombination
Pol: Polymerase
SIF: stumpy induction factor
VSG: variant surface glycoprotein
